# Motivational Interview Techniques and the Effectiveness of Intervention Programs With Perpetrators of Intimate Partner Violence: A Systematic Review

**DOI:** 10.1177/15248380221111472

**Published:** 2022-07-06

**Authors:** Teresa Pinto e Silva, Olga Cunha, Sónia Caridade

**Affiliations:** 1 70990 University Fernando Pessoa (UFP), Porto, Portugal; 2 HEI-Lab: Digital Human-Environment Interaction Lab, 386292 Universidade Lusófona Do Porto, Portugal; 3 Psychology Research Center, School of Psychology, University of Minho, 219951 Campus de Gualtar, Braga, Portugal

**Keywords:** intimate partner violence, perpetrators, intervention program, motivational interviewing techniques

## Abstract

Intimate Partner Violence (IPV) is widely recognized as a severe public health issue. Perpetrators’ Intervention Programs (PIPs) have been essential to prevent recidivism, and the incorporation of Motivational Interview Techniques (MIT) has shown to be an added value in this area. **Objective:** The present systematic review aims to analyze the incorporation of MIT (i.e., pre-treatment, isolated treatment, and conjoined with PIPs) in interventions with IPV perpetrators and its potential impact on their behavior and attitudes regarding motivation for change and treatment compliance. **Method:** The following research equation was used: “Intimate Partner Violence” AND (“Perpetrator” OR “Batterer” OR “Offender”) AND (“Motivation” OR “Motivational Interview”) AND (“Intervention” OR “Intervention Program” OR “Batterer Intervention Program”) AND (“Effectiveness OR “Program Effectiveness”); in four separate databases: PubMed, PsycINFO, Science Direct, and EBSCO. Studies in English, Portuguese, and Spanish were included, and 15 were identified according to the defined inclusion criteria. **Results:** Studies demonstrated that MIT increases attendance rates, treatment adherence, motivation for change, and behavioral and attitudinal outcomes. More specifically, MIT showed greater effectiveness among participants with low readiness to change and in the early stages of change. **Conclusion:** This systematic review corroborates the importance of incorporating MIT in PIPs to improve intervention efficacy.

## Introduction

Intimate partner violence (IPV) is one of the most common forms of violence against women (VAW), being women the ones who bear the overwhelming global burden of IPV (World Health Organization [WHO], 2021). IPV is not a recent problem; however, it only began to gain visibility in the 70s, almost simultaneously with the emergence of the first perpetrators’ intervention programs (PIPs), mainly in the United States and the United Kingdom (Caridade & Sani, 2018). The development of PIPs is based on the argument that punitive strategies per se are insufficiently effective with perpetrators of IPV, as the recidivism rate remains high. Without a specialized intervention, the likelihood of men returning to violent and abusive behaviors in their current or future relationships is relatively high (Manita & Matias, 2016).

Despite the reduced structuring characterizing the first intervention initiatives with perpetrators, these evolved towards more structured treatment programs, incorporating psychoeducational models (e.g., Duluth) and cognitive-behavioral techniques (Butters et al., 2021), which were the subject of most research on the treatment of IPV perpetrators (Arce et al., 2020; Arias et al., 2013). These two models have been the ones most used to treat IPV perpetrators (Cannon et al., 2016). Conceptualizing IPV as a product of patriarchy or male socialization, in which the perpetrator seeks to gain power and control from his partner (Pender, 2012), the Duluth model emerged as one of the first responses to the treatment of IPV perpetrators (Babcock et al., 2004). This model combines a gender perspective with a psychoeducational approach to modify male perpetrators’ attitudes towards women and promote egalitarian relationships (Butters et al., 2021; Cannon et al., 2016), using different strategies (e.g., role-plays, individualized action plans, video enactments, spreadsheets and records, figures that contrast equality vs. power and control) (Pender, 2012). Cognitive-behavioral therapy (CBT) emerges as an alternative to the Duluth model, constituting a therapeutic modality that seeks to change useless thoughts and behaviors and promote skills to improve perpetrators’ functioning (Butters et al., 2021). More specifically, the predominant therapeutic objective of the intervention with IPV perpetrators is to stop the abusive behaviors and decrease the levels of anger and depression, promoting changes in attitudes towards victims, and adopting non-disruptive behaviors (Cunha et al., 2022; Cunha & Gonçalves, 2015; Illescas, 2008). According to Arce et al. (2020), intervention programs for IPV perpetrators should be based on a long cognitive-behavioral approach, considering its efficacy.

Despite the high dissemination of PIPs, their effectiveness in reducing future incidents of IPV remains uncertain and controversial. Although several studies have revealed positive effects for perpetrators who complete intervention programs (e.g., Cunha & Gonçalves, 2015; Lauch et al., 2017; Lila et al., 2020), results from meta-analyses (e.g., Arias et al., 2013; Babcock et al., 2004; Cheng et al., 2019; Travers et al., 2021) are mixed. While some studies concluded that PIPs (both Duluth and CBT interventions) had minor effects on IPV and recidivism rates reduction (e.g., Arias et al., 2013; Babcock et al., 2004; Feder & Wilson, 2005; Travers et al., 2021); others claimed a significant effect of PIPs in violence reduction (Karakurt et al., 2019); and others revealed an effective decrease on IPV recidivism when reported by the criminal justice system, but not when reported by the victim (Cheng et al., 2019). One of the main problems related to PIPs that may interfere with its efficacy is the high dropout rates (e.g., Cunha & Gonçalves, 2014; Jewell & Wormith, 2010). The lack of consideration for the perpetrators’ readiness and motivation for change has been identified as the main reasons for these high dropout rates (Lila et al., 2018). Thus, despite the inconsistencies regarding PIPs’ effectiveness, there is a consensus on the need to improve PIPs to increase their efficacy (Lila et al., 2018). Therefore, alternative strategies and techniques for the treatment of IPV perpetrators have been identified (Butters et al., 2021). Motivational interview techniques (MIT), as a single intervention and a complement to other treatments, are one example of such strategies. Indeed, MIT shows promise in improving the efficacy of PIPs and reducing the dropout rate (Santirso et al., 2020a), at least for those in the earlier stages of change (i.e., pre-contemplation and contemplation; Butters et al., 2021). Thus, greater attention and consideration for each perpetrator's individual needs, characteristics, and readiness to change would help promote motivation for change, treatment compliance, and reduction in the dropout rate (Butters et al., 2021; Lee et al., 2004; Lila et al., 2018).

Motivational Interviewing (MI) aims to promote the individual’s involvement in treatment and increase motivation for change, being the client the only one responsible for his/her change (Miller & Rollnick, 2012). This approach is client-focused and begins with the establishment of a collaborative therapeutic alliance—rapport (Cunha, 2016; Miller & Rollnick, 2012), that is, a connection between the interviewer and the interviewee (Vallano et al., 2015). MI is based on the transtheoretical model of change of Prochaska and DiClemente (1997). It assumes that, until they achieve change, all individuals go through a series of stages: (i) pre-contemplation, in which the individual denies the existence of a problem, minimizes it or attributes it to external causes; (ii) contemplation, where participants begin to understand the existence of a problem but are not yet involved with the change; (iii) preparation for action, where the individual begins to consider more conscientiously ways to change his behavior; (iv) action, where the individual is already actively involved in his/her change; and (v) maintenance, the last stage and the one where the individual intends that the problem does not arise again. The therapist helps the client to progress through these stages toward change, and, simultaneously, the individual changes his behaviors (Cunha, 2016). Throughout their progression through different stages, positive changes become more stable and internalized. However, strategies for one phase may not be effective for another phase and may even be counterproductive (Wong et al., 2007). As such, MI is governed by five basic principles: express empathy, develop discrepancy and dissonance between client behavior and their goals and values, avoid argumentation and confrontative strategies, reduce resistance, and reinforce self-efficacy by promoting the client's confidence that he/she has the necessary skills to change (Austin et al., 2011). MI may be used in three distinct ways: (i) as a single therapy; (ii) combined with other treatments, aiming to improve its benefits; or (iii) as an intervention before the main treatment to increase commitment to the subsequent treatment (Soleymani et al., 2018).

Different studies have investigated the efficacy of MIT with perpetrators of IPV. For example, Soleymani et al. (2018) analyzed the efficacy of MIT as a pre-treatment intervention to promote commitment to treatment for men referred to PIPs. The authors examined whether the studies included MIT and whether MIT was consistent with Zuckoff et al.’s (2015) recommendations, that is, MIT should not only consider the motivation for changing the behavior but also take into consideration additional factors that might influence engagement in treatment (Soleymani et al., 2018). Soleymani et al. (2018) concluded that MIT could positively affect commitment to intervention programs; however, none of the studies reviewed considered MIT according to the conceptualization of Zuckoff et al. (2015). In the studies analyzed, MIT was considered as a technique to modify violent behavior and not to promote commitment to treatment. In addition, a meta-analysis conducted by McMurran (2009) aimed to systematically review the impact of MI or MIT on offender populations. It established that MI had been frequently evaluated with substance misusing offenders; however, other applications, such as IPV perpetrators, drunk drivers, and general offenders, were also noticeable. MI was used to enhance retention and engagement in treatment, improve motivation for change, and change behavior. Despite its pertinence, this study was held more than 10 years ago. More recently, Santirso et al. (2020a) performed a meta-analysis of randomized controlled trials (RCTs) of interventions for IPV perpetrators that incorporated MIT, published between 1983 and 2018. Results indicated that IPV interventions incorporating MIT were significantly more effective in increasing the intervention dose and reducing the dropout rate than interventions without MIT. Although this meta-analysis only included RCTs, which was assumed as a strength, it might be simultaneously perceived as a limitation given the difficulties and downsides of using RCTs in IPV perpetrators’ treatment, as supported in another review (Lilley-Walker et al., 2018). Indeed, conducting RCTs with IPV perpetrators is a challenge that is not always easy to overcome given the specificities of the sample, which leads researchers to consider the use of other designs than RCTs (Lilley-Walker et al., 2018). Thus, regarding the diversity of research designs, the number of non-experimental designs (Lilley-Walker et al., 2018), and the fact that both RCTs and less robust designs offer some directions for both research and practice (McMurran, 2009), in this systematic review we used an inclusive methodological approach. In this sense, based on different research designs, we aimed to analyze the incorporation of MIT (i.e., pre-treatment, isolated treatment, and conjoined with PIPs) in interventions with IPV perpetrators and their potential impact on perpetrators’ behavior and attitudes, motivation/readiness for change, and treatment adherence/dropout rates. More specifically, we aim to (i) develop a descriptive overview of the research on the efficacy of MIT with IPV perpetrators and to reveal the most relevant research trends on this subject; (ii) understand the relevance of MIT and how is it being approached and analyzed within the existing PIPs, and (iii) to understand whether the perpetrators’ stage of change and readiness to change might influence MIT’s outcomes.

## Methodology

The present systematic review was conducted according to the Preferred Reporting Items for Systematic Reviews and Meta-Analyses (PRISMA) guidelines (Moher et al., 2009).

### Eligibility Criteria

The following criteria were used to determine whether studies were eligible for inclusion: (i) sample of male participants; (ii) sample of adult participants convicted for IPV; (iii) incorporation of MIT in the treatment; and (iv) published articles written in English, Spanish, or Portuguese.

### Search Strategies

Initially, we defined different keywords and their combination, creating the following search equation: “Intimate Partner Violence” AND (“Perpetrator” OR “Batterer” OR “Offender”) AND (“Motivation” OR “Motivational Interview”) AND (“Intervention” OR “Intervention Program” OR “Batterer Intervention Program”) AND (“Effectiveness OR “Program Effectiveness”). This combination of keywords was used to run the search in several electronic databases: PubMed, PsycINFO, Science Direct, and EBSCO. We limited our search to titles and abstracts, and manuscripts written in English, Portuguese, and Spanish. Including publications in Portuguese and Spanish was related to the emergence and growth in the number of BIPs in Portugal and Spain (Ferrer-Perez et al., 2016). The search was carried out between September 2021 and January 2022. We also screened the reference lists of reviews/meta-analyses on the subject (McMurran, 2009; Santirso et al., 2020a; Soleymani et al., 2018) to verify the existence of additional references not identified through our database search. Finally, we contacted authors in the field to request additional references, which is why we have incorporated a book chapter from an RCT (Lila et al., 2020).

### Data Extraction

**Figure 1.**
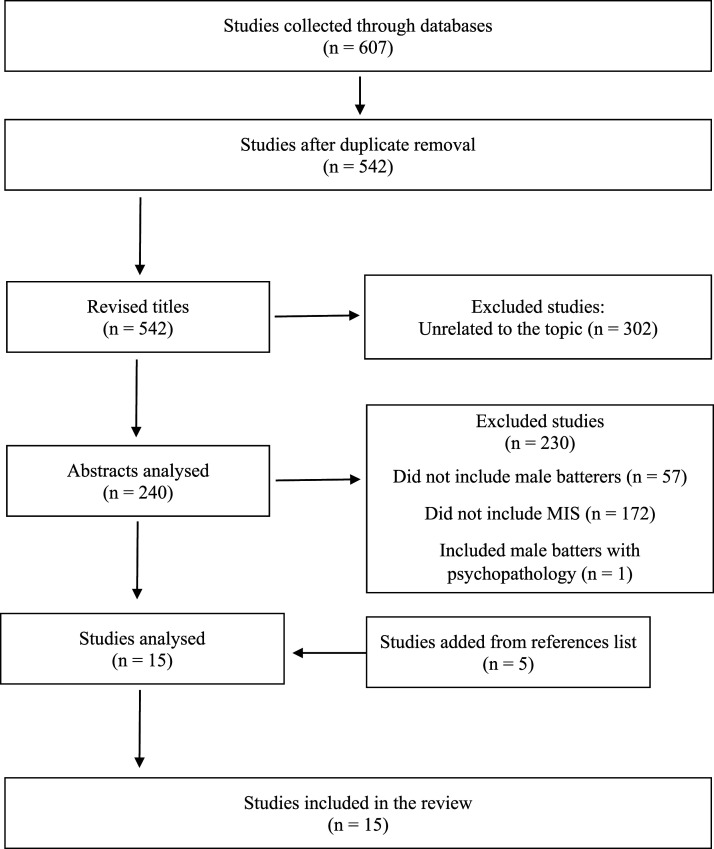
Flowchart of selection of studies**.** Excluded studies (n = 230) Did not include male batterers (n = 57) Did not include MIS (n = 172) Included male batters with psychopathology (n = 1).

Reference data were retrieved, and duplicates were subsequently eliminated. Titles and abstracts were then read to determine if the articles met the inclusion criteria. Articles that met the inclusion criteria through screening the title and abstract were retrieved and fully read to reach a final decision (Figure 1).

### Coding Procedures

A codebook was developed to extract data from all the included manuscripts, including the following key characteristics: reference information (e.g., authors, year); studies' characteristics (e.g., location, objectives); sampling characteristics (e.g., sampling frame, sampling procedures, response rate); samples’ characteristics (e.g., size, age, sex, ethnicity/race); design characteristics (e.g., design type, length of follow-up); intervention characteristics (e.g., setting, EM modality, number of sessions or hours, complementary intervention); measurement characteristics (e.g., assessment measures, assessment of recidivism); intervention’s results (e.g., dropout/completion rate; efficacy).

All articles were independently coded by the first and the last authors. A third reviewer verified all data and disagreements were resolved through discussion.

### Methodological Quality Analysis

The Mixed Methods Appraisal Tool (MMAT; Hong et al., 2018) was used to assess the methodological quality of all studies included. This tool proved essential to limit the bias in synthesizing evidence. The MMAT starts with two screening questions (e.g., “Are there clear research questions?”; “Do the collected data allow to address the research questions?”). Five items are considered to assess the methodological quality of studies, depending on their quantitative design (e.g., randomized controlled trials, non-randomized trials). Each of the criteria is classified as “yes”, “no,” or “don't know.” A more detailed analysis of the classifications of each criterion to obtain more information about the weaknesses of the study was carried out and later used in the discussion of the agreement between coders. Two authors independently assessed the studies' methodological quality. Disagreements were resolved through discussion with another author.

## Results

The main results are displayed in Tables 1 and 2

**Table 1.** Characteristics of Studies.

Author/s, year of publication, and location	Sampling frame	Sample		Objectives
		Size	Age	
Kistenmacher and Weiss (2008); USA	Justice system	Ntotal = 252: BAI (*N* = 123) SBI (*N* = 129)	BAI: M= 31.5; SD = 9.6 SBI: M = 31.6; SD : 9.9	Evaluate the effectiveness of MI in changing the way batterers think about their violent behavior
Musser et al. (2008); USA	Community	*N* = 211	M = 34.6; SD = 10.77	Assess the effects of a pre-group motivational intervention for IPV perpetrators
Alexander et al. (2010); USA	Justice system	*N* = 42: Individual CBT (*N* = 21); group CBT (*N* = 21)	ICBT: M= 36.86; SD = 7.12 GCBT: M = 31.90; SD = 6.07	Comparison between a stages-of-change motivational interviewing (SOCMI) treatment approach and a standard cognitive-behavioral therapy gender reeducation (CBTGR) approach
Scott et al. (2011) Canada	Justice system	*N* = 160	M = 40.66; SD = 11.54	Presentation of results of a motivation improvement intervention with highly resistant batterers
Connors et al. (2012); Canada	Justice system	*N* = 228: MET (*N* = 110); AE (*N* = 118)	Not specified	Evaluation of the moderate intensity family violence prevention program (MIFVPP)
Murphy et al. (2012); USA	Justice system	*N* = 93: SBIP (*N* = 40) SBIP + IMP (*N* = 53)	SBIP: M = 41.80; SD = 10.69 SBIP + IMP:M = 39.75; SD = 10.19	Analyze predictions about the differential response to a two-session MI for partner-violent men, which was compared to a structured Intake (SI) control
Crane and Eckhardt (2013); USA	Justice system	Study 1: *N* = 123 Study 2: *N* = 93 Study 3: *N* = 120	M = 40/42	Evaluation of the effectiveness of a single brief motivational interview session (BMI) within batterers intervention programs (BIPs)
Stuart, Shorey, Moore, Ramsey, Kahler, O'Farreall, Strong, Temple & Monti (2013); USA	Justice system	*N* = 153	M = 40.73 SD = 11.99	Examined whether adding adjunctive alcohol intervention to batterer intervention reduced the likelihood of substance use and violence relative to batterer intervention alone
Zalmanowitz et al. (2013) Canada	Justice system	*N* = 252: BAI (*N* = 123) SBI (*N* = 129)	BAI: M= 31.5; SD = 9.6 SBI: M = 31.6; SD : 9.9	Investigate the impact of MI and the state of change in a globally functioning self-report instrument (outcome questionnaire [OQ 45.2])
Murphy et al. (2017) USA	Community	*N* = 211	M = 34.6; SD = 10.77	Investigate the effectiveness of an individual treatment approach integrating MIT with CBT in comparison with a standardized group CBT for perpetrators of IPV.
Lila, Gracia & Catalá-Miñana (2018); Spain	Justice system	*N* = 42: Individual CBT (*N* = 21) Group CBT (*N* = 21)	ICBT: M= 36.86; SD = 7.12 GCBT: M = 31.90; SD = 6.07	Examine whether adding an individualized motivational plan (IMP) to a standard BIP (SBIP) increased intervention effectiveness relative to BIP alone
Murphy et al. (2018); USA	Community	*N* = 160	M = 40.66; SD = 11.54	Examine the efficacy of brief alcohol intervention in the context of community-based treatment for partner violence
Romero-Martínez et al. (2019) Spain	Justice system	*N* = 228: MET (*N* = 110) AE (*N* = 118)	Not specified	Compare the effectiveness of two different modalities of IPV intervention programmes (standard Batterer intervention programs [SBIPs] vs. SBIP + individualized motivational plan [IMP]) in promoting empathic improvements after both interventions
Lila et al. (2020) Spain	Justice system	*N* = 93: SBIP (*N* = 40) SBIP + IMP (*N* = 53)	SBIP: M = 41.80; SD = 10.69 SBIP + IMP: M = 39.75; SD = 10.19	Analyze whether the inclusion of MIT increases the effectiveness of BIPs, compared to standard programs
Santirso, Lila and Gracia (2020); Spain	Justice system	Study 1: *N* = 123 Study 2: *N* = 93 Study 3: *N* = 120	M = 40/42	Explore whether adding an IMP to a standard BIP improved the participant-facilitator working alliance and participants’ protherapeutic behaviors

Measurement							
	Setting	Design type	Length of follow-up	EM modality	Number of sessions or hours	Complementary intervention	Assessment measures
Kistenmacher and Weiss (2008)	Community	RCT	Not specified	Individual	2 sessions (50–60 minutes)	No	Self-report
							• CTS (Straus, 1979)
							• BAI-R (Gudjonsson & Singh, 1989)
							• SOCQ (McConnaughy et al. 1983)
Musser et al. (2008)	Community	RCT	6 months	Individual	2 sessions (45 minutes)	Cognitive-behavioral group intervention	Self-report
							• SIRC (Begun et al., 2003)
							• ACRS (Bryant et al., 1986)
							• CTS (Straus, 1979)
							• CTS2 (Straus et al., 1996)
							• State-trait anger expression inventory (Spielberger, 1988)
							• Dyadic adjustment Scale (Spanier, 1976)
							Other source
							• WAI (Horvath & Greenberg, 1989)
							• Observational coding of in-session behavior
Alexander et al. (2010)	Community	RCT	6 and 12 months	Group	26 sessions	No	Self-report
							• CTS2 (Straus, Hamby, Boney-McCoy, & Sugarman, 1996)
							• URICA (McConnaughy, et al., 1989)
							Other source: Victim partner data collection – CTS2
Scott et al. (2011)	Community	Quasi-experimental trial	Not specified	Group	6 sessions	(Pro)Feminist group intervention	Self-report
							• Resistance screening
							• ACT (Baxter & Tweedale, 1995)
							Other source: Counselor-rated success
Connors et al. (2012)	Prison	Quantitative no randomized	Not specified	Both (individual and group)	Group – 29 sessions (3 hours) Individual – 3 sessions	No	Self-report
							• IRS (Hupka & Rusch, 2001)
							• AQ-R (correctional Service Canada, 2001)
							• ARI (Boer, 1993)
							• OSRC (correctional Service Canada, 2001)
							Interview: Domestic violence vignettes (DVVs)
							Other source: Facilitator structured ratings
Murphy et al. (2012)	Community	Quantitative no randomized	6 months	Individual	2 sessions (45 minutes)	Cognitive-behavioral group intervention	Self-report
							• STAXI (Spielberger, 1988)
							• SAH (Begun et al., 2003)
							• WAI (Horvath & Greenberg, 1989)
							• ACRS (Bryant et al., 1999; Primakoff et al., 1986)
							Other source: Posttreatment partner assault – CTS2
Crane and Eckhardt (2013)	Community	RCT	6 months	Individual	1 session (45–55 minutes)	Yes (not specified)	Self-report
							• CTS2 (Straus et al., 1996)
							• DAS-4 (Sabourin, Valois, Lussier, 2005)
							• SAH (Begun et al., 2003)
Stuart et al. (2013)	Community	RCT	Quarterly follow-ups for 1 year	Individual	1 session (90 minutes)	Yes (not specified)	Self-report
							• CTS2 (Straus et al., 1996)
Zalmanowitz et al. (2013); Canada	Community	Quasi-experimental	Not specified	Individual	2 sessions	(Pro)Feminist group intervention	Self-report
							• OQ (Lambert et al., 1996)
							• URICA (McConnaughy et al., 1983, 1989)
							• URICA-DV (Levesque et al., 2000)
Murphy et al. (2017)	Community	RCT	Quarterly follow-ups for 1 year	Individual	20 sessions (1 hour)	No	Self-report
							• CTS2 (Straus et al., 1996)
							• MMEA (Murphy et al., 1999)
							• DAS (Spanier, 1976)
							Official records: Criminal justice outcomes
							Other source: Participant communication difficulties – VPC (Haynes et al., 1984)
Lila et al. (2018)	Community	RCT	6 months	Both (individual and group)	Individual – 5 sessions (1 hour) Group – 3 sessions	Cognitive-behavioral group intervention	Self-report
							• CTS2 (Straus et al., 1996)
							Official records: Recidivism
							Other source
							• Risk of recidivism assessment by therapists
							• Stage of change – rated by therapists
Murphy et al. (2018)	Community	RCT	Quarterly follow-ups for 1 year	Individual	4 weeks	No	Self-report
							• URICA (DiClemente & Hughes, 1990)
							• TLFB (Sobell & Sobell, 1996)
Romero-Martínez et al. (2019)	Community	RCT	9 months	Individual	3 sessions	Cognitive-behavioral group intervention	Self-report
							• IRI – Interpersonal reactivity index
							Other source: Neuropsychological test - reading the mind in the
							Eyes (eyes test)
Lila et al. (2020)	Community	RCT	6 months	Individual	5 sessions	Cognitive-behavioral group intervention	Self-report
							• CTS-2 (Straus et al., 1996)
							• SARA (Kropp et al., 1995)
							• IRI (Davis, 1983)
							• WAI-O-S (Tichenor & Hill, 1989; adaptación de Santirso et al., 2018)
							Official records: Recidivism
							Other source
							• Neuropsychological test - reading the mind in the eyes (eyes test)
							• Stage of change – rated by therapists
Santirso et al. (2020)	Community	RCT	6 months	Individual	5 sessions	Cognitive-behavioral group intervention	Self-report
							• WAI-O-S (Tichenor y Hill, 1989; adaptación de Santirso et al., 2018)
							Other source: Observational coding of protherapeutic group behavior

**Table 2.** Main Results of the Studies (*n* = 15) included in the Systematic Review.

Author/s, year of publication	Main results
Kistenmacher and Weiss (2008)	• 85% of the participants completed the two phases of the study – 94% of the control group and 75% of the group of participants submitted to MI.
	• Five participants (15%) did drop out of the study
	• The group submitted to MI demonstrated an evolution in thinking about the change in its behavior, unlike the control group
	• The MI group demonstrated lower outsourcing of guilt and greater recognition of the responsibility of its behavior, unlike the control group
	• MI group demonstrated a pre-to-post increase in the action and contemplation stage, while the control group showed a decrease in those same stages
	• The control group demonstrated a pre-to-post decrease in the pre-contemplation stage, and the MI group showed a slight increase in pre-contemplation
Musser et al. (2008)	• Two sessions of MI significantly enhanced treatment engagement and help-seeking behavior but did not significantly alter treatment session attendance or subjective self-reports of readiness to change
	• MI participants displayed more constructive behavior in session when they began attending subsequent group CBT, articulating the greater perceived value of treatment and assuming more personal responsibility for their abusive behavior
	• MI participants completed substantially more of the assigned CBT homework than SI participants
	• MI participants had a more robust working alliance than SI participants
	• In addition, more of the MI participants reported that they had obtained help from sources outside of the domestic violence program during treatment
Alexander et al. (2010)	• Significantly fewer partners of men assigned to the SOCMI treatment condition as opposed to the CBTGR condition reported having experienced physical aggression at follow-up
	• The two treatment conditions did not differ concerning partner follow-up reports of psychological aggression
	• Men who were less ready to change at intake were more likely to benefit from the SOCMI condition, while men who were more ready to change at intake were more likely to benefit from the standard CBTGR condition
	• Differential growth in men's self-reported readiness to change was not observed between treatment conditions
Scott et al. (2011)	• Resistant men reported significantly lower overall motivation scores than men classified as non-resistant. They displayed significantly more negative attitudes across 4 of the five subscales: Treatment motivation and perceived need for treatment, perceptions of treatment and the program, perceptions of staff, and optimism towards treatment outcome
	• No significant differences were observed in self-disclosure comfort levels between identified resistant and non-resistant clients
	• Resistant batterers who participated in the specialized intervention concluded it with a higher commitment rate than the remaining participants
	• A higher dropout rate in resistant participants integrated into the standard intervention (53.5%), followed by non-resistant participants (38.9%) and resistant participants submitted to specialized intervention (15.8%)
	• Higher completion rate in resistant clients who attended the MET group (84.2%), followed by resistant clients in the standard intervention (46.5%) and non-resistant clients (61.1%)
Connors et al. (2012)	• 15% of the participants did not complete the program
	• Improve motivation for change and a consequent improvement in results
	• Positive change in attitudes, a reduction in feelings of jealousy, anger, and dependency, an increase in their acceptance of responsibility, an improvement in their ability to dispute their cognitive distortions regarding their violence, and their ability to engage in perspective-taking and dealing with conflict
	• The initial facilitator ratings of motivation to change for the 15% who did not complete was statistically lower than those who did complete the program
Murphy et al. (2012)	• 69% of the individuals in MI condition (27 of 39) versus 31% in the SI condition (12 of 39) reported being in a different stage of change after the intake process than before the intake process
	• There was very little association between initial contemplation scale scores and subsequent working alliance ratings among those in the SI condition, whereas, in the MI condition, those reporting higher contemplation of change developed stronger working alliances
Crane and Eckhardt (2013)	• There were no significant differences in recidivism between intervention conditions on aggressive, non-aggressive, and non-recidivism outcomes
	• A greater percentage of BME condition participants (72.9%) had either successfully completed their BIP or remained in good standing 6 months post-adjudication, relative to control participants (50.0%)
	• BME participants were more likely to attend the initial 6 BIP sessions and consistently, but not significantly, exhibit higher attendance rates throughout the remaining 20 sessions relative to control participants
	• Males high in readiness to change were equally likely to comply with treatment regardless of BME (64.0%) or control (60.0%) condition. Readiness to change predicted the number of sessions attended
Stuart et al. (2013)	• Men receiving SBP + BAI reported consuming fewer drinks per drinking day than men in SBP for the first 3 months, although this difference faded by the 6-month follow-up. Men receiving SBP + BAI reported using alcohol on significantly fewer days for 6 months following the brief intervention, relative to men in SBP, although this difference faded by 12 months
	• In terms of IPV, men receiving SBP + BAI reported less frequent perpetration of severe psychological aggression and violence causing injuries to their partners at 3- and 6-month follow-up, relative to SBP, with differences weakening at 12 months. Similarly, men in SBP + BAI reported less frequent severe physical violence perpetration at 3-month follow-up, relative to SBP, with differences fading over time
	• Men receiving SBP + BAI reported significantly greater abstinence at 3- and 6-month follow-up, but not 12-month follow-up
	• There were no significant differences in physical IPV between men receiving SBP and men receiving SBP + BAI. Men receiving SBP + BAI reported less severe physical aggression at 3-month, but not 6- or 12-month follow-up. Men receiving SBP + BAI reported less severe psychological aggression and fewer injuries to partners at 3- and 6-month follow-up, with differences fading by 12 months
	• Men with a history of intimate partner violence and hazardous drinking who received a batterer intervention plus an alcohol intervention initially showed improved alcohol and violence outcomes, but improvements faded by 12 months
Zalmanowitz et al. (2013); Canada	• Men who did not receive the MI (reference group) had higher OQ scores at the intercept. Their average score was 38.97 compared to 33.21 for men who received the MI.
	• Participants who were not submitted to MI obtained higher scores in the OQ, showing higher levels of distress
	• Participants submitted to MI reported less distress in the OQ regardless of the state of change in which they were
Murphy et al. (2017)	• 90% of the participants in the ICBT condition and 62% of those in the GCBT condition completed the study
	• For the intent-to-treat sample, treatment uptake was higher in the ICBT condition, as evidenced by attendance of one or more treatment sessions (100% in ICBT; 71% in GCBT)
	• Among those who attended at least one treatment session, completion of a credible dose was similar across conditions (90% in ICBT; 87% in GCBT), but voluntary uptake of sessions beyond the core treatment was higher in ICBT (33%) than GCBT (7%)
	• Partner reports of CTS2 psychological aggression declined over time in GCBT but increased in ICBT.
	• Group CBT produced outcomes consistently equal to, or better than, ICBT.
	• Treatment uptake, attendance, and voluntary continuation were higher in ICBT than GCBT.
Lila et al. (2018)	• Thirty-seven participants (23%) did not complete the study
	• Findings indicated that the SBIP + IMP participants received significantly more intervention dose, finished the intervention in a more advanced stage of change, reported less physical violence after treatment, and had a higher reduction in recidivism risk than SBIP participants
	• The analyses of the sample characteristics of completers and non-completers revealed differences in income and risk of recidivism. The completers group reported higher levels of income and lower levels of risk of recidivism at baseline
	• Official recidivism data showed that 7.5% (*n* = 12) of the participants in the total sample were rearrested on one or more occasions after treatment. No significant differences were found in recidivism rates between experimental and control groups
	• Participants in the SBIP + IMP condition reported less physical violence and had a higher reduction in recidivism risk informed by therapists
Murphy et al. (2018)	• MET participants displayed greater acknowledgment of problems with alcohol than AE participants
	• Significant changes from baseline across treatment conditions were observed for percent days of alcohol abstinence, heavy drinking, illicit drug use, and partner violence
	• 90% of the participants in the MET condition and 85.6% of those in the AE condition completed the study
Romero-Martínez et al. (2019); Lila et al. (2020)	• After analyzing each group separately, within-group comparisons only revealed significant effects for ‘time’ on the eyes test in IPV perpetrators who received the SBIP + IMP. After the intervention program, this group of IPV perpetrators presented higher eyes test scores. SBIP + IMP participants evidenced greater empathic changes/improvement
	• Results indicated that participants in the experimental condition (BIP + PMI) reported less physical violence and greater risk reduction of recidivism reported by the coordinators
	• Although in the BIP + PMI condition a significant reduction in self-reported psychological violence was found at the end of the intervention, no significant differences were found between both conditions in this variable
	• Participants in the experimental condition presented higher scores in therapeutic alliance than those assigned to the control condition, regardless of the intervention time
	• Participants in the experimental condition completed the intervention at a more advanced stage of change than participants in the control condition
	• In terms of emotional decoding and empathy, only participants in the experimental condition improved their ability to decode emotions and perspective-taking
	• 20% of the participants in the MIT condition and 26.25% of the control condition did not complete the study
Santirso, Lila, and Gracia (2020)	• Participants who received SBIP + IMP intervention showed higher general working alliance than those who received SBIP intervention, regardless of intervention moment
	• Participants who received SBIP + IMP intervention showed higher responsibility for abuse early and late in the intervention than those who received SBIP.
	• Participants who received SBIP + IMP condition had significantly higher participant role behavior scores and tended to show higher participant role behaviors scores late in treatment than those who received SBIP only
	• Responsibility for abuse and participant role behaviors significantly increased throughout intervention in participants who received SBIP, but not those in SBIP + IMP condition
	• Fifty-four participants who received SBIP + IMP and 50 who received SBIP intervention completed the study

*Notes.* RCT = Randomized Controlled Trail/ Randomized Clinical Trial; CBT = Cognitive-behavioral therapy; SOCMI = stages-of-change motivational interviewing; BAI = brief alcohol intervention; SBI = standard batterer intervention; SBIP = standard batterer intervention Program; MET = motivational enhancement therapy; AE = alcohol education; IMP = individualized motivational plan; CTS = Conflict Tactics Scale Form N; CTS2 = Conflict Tactics Scales-Revised; BAI-R = revised gudjonsson Blame attribution inventory; SOCQ = stages of change questionnaire; SIRC = Safe-at-Home Instrument for Assessing Readiness to Change Intimate Partner Violence; ACRS = assignment Compliance rating scale; WAI = working Alliance inventory; URICA = university of rhode island change assessment; URICA-DV = University of Rhode Island Change Assessment—Domestic Violence; ACT = attitudes towards correctional treatment; IRS = interpersonal relationship scale; AQ-R = Aggression Questionnaire–Revised; ARI = abusive relationship inventory; OSRC = Offenders’ self-rated readiness to change; STAXI = State-Trait Anger Expression Inventory; SAH = Safe-At-Home Instrument; DAS-4 = Dyadic Adjustment Scale – four; OQ = outcome questionnaire; MMEA = multidimensional measure of emotional abuse; DAS = dyadic adjustment scale; VPC = spouse verbal problem checklist; TLFB = Time-Line Follow-Back Interview; IRI = interpersonal reactivity index; SARA = spousal assault risk assessment; WAI-O-S = Working Alliance Inventory Shortened Observer-rated version; M = mean; SD.= standard deviation

### Included Studies

Our electronic database search yielded 607 references; 65 were duplicates and consequently removed. Thus, 542 titles were screened to assess eligibility and consequently 302 were excluded because they were unrelated to the topic. Then, 240 abstracts were analyzed. Of those, 230 were excluded because they did not meet the eligibility criteria. The main reasons for exclusion were: (i) did not include male convicted perpetrators; (ii) did not include MIT; and (iii) included male perpetrators with diagnosed psychopathology. Five manuscripts were added through the hand search of reference lists. As a result, 34 manuscripts were fully read, and 19 were excluded since they did not meet the eligibility criteria: (i) did not include male convicted perpetrators (*n* = 6; e.g., Woodin & O’Leary, 2010); (ii) did not include MIT (*n* = 8; e.g., Ramírez et al., 2013); (iii) included male perpetrators with diagnosed psychopathology (*n* = 4;e.g., Kraanen et al., 2013) and one study did not meet any criteria. So, 15 studies were included in the systematic review and marked with an “*” in the references’ section.

### Quality Assessment

Among the included articles, most were designed as Randomized Control Trials (*n* = 11), with only four studies using different designs: Connors et al., 2012 and Murphy et al. (2017) conducted a quantitative non-randomized study, and Scott et al. (2011) and Zalmanowitz et al. (2013) carried out a quasi-experimental study.

Of the 15 studies, eight showed all the criteria of excellent (Alexander et al., 2010; Kistenmacher & Weiss, 2008; Lila et al., 2018; Murphy et al., 2017, 2018; Romero-Martínez et al., 2019; Santirso et al., 2020b; Stuart et al., 2013), six presented four out of five criteria of excellent (Connors et al., 2012; Crane & Eckhardt, 2013; Lila et al., 2020; Musser et al., 2008; Scott et al., 2011; Zalmanowitz et al., 2013) and one showed three out of five criteria (Murphy et al., 2012).

### Reference Information and Study’s Characteristics

The year of publication of the articles varied between 2008 (Kistenmacher & Weiss, 2008; Musser et al., 2008) and 2020 (Lila et al., 2020; Santirso et al., 2020b). The year with the highest number of publications was 2013 (*n* = 3), followed by 2020 (*n* = 2), 2018 (*n* = 2), 2012 (*n* = 2), and 2008 (*n* = 2). Most of the studies were conducted in America, notably in the United States of America (USA; *n* = 8; Alexander et al., 2010; Crane & Eckhardt, 2013; Kistenmacher and Weiss, 2008; Murphy et al., 2012, 2017, 2018; Musser et al., 2008; Stuart et al., 2013) and Canada (*n* = 3; Connors et al., 2012; Scott et al., 2011; Zalmanowitz et al., 2013). In Europe, four studies were conducted, more precisely in Spain (Lila et al., 2018, 2020; Romero-Martínez et al., 2019; Santirso et al., 2020b).

Most manuscripts were journal articles (*n* = 14), apart from the one conducted by Lila et al. (2020), consisting of a book chapter.

### Sample Characteristics

The sample size of the studies ranged between 33 (Kistenmacher and Weiss, 2008) and 528 (Alexander et al., 2010) male perpetrators of IPV. The mean age of the participants ranged between 31.5 (Stuart et al., 2013) and 41.80 (Romero-Martínez et al., 2019). Concerning participants’ ethnicity/race, most of them were White/Caucasian, ranging between 2% and 72.1%; followed by African American, ranging between 0% and 47.6%; Latino/Hispanic, ranging between 2% and 15.4%; and Asian, ranging between 0% and 4.8%.

The selected studies had three main objectives: to assess the effectiveness of MI (*n* = 10; Alexander et al., 2010; Crane &Eckhardt, 2013; Kistenmacher &Weiss, 2008; Lila et al., 2018, 2020; Murphy et al., 2012, 2017, 2018; Musser et al., 2008; Scott et al., 2011); to validate instruments/programs with the integration of MIT (*n* = 2; Connors et al., 2012; Zalmanowitz et al., 2013); and to analyze specific variables, such as alcohol abuse, empathy and therapeutic alliance, after the implementation of MI (*n* = 3; Romero-Martínez et al., 2019; Santirso et al., 2020b; Stuart et al., 2013).

Two sampling procedures were identified: Random Sampling (*n* = 7) and Total Sampling (*n* = 8). Only two studies indicated values concerning response and retention rates: Kistenmacher and Weiss (2008) identified a response rate of 27% and a retention rate of 73%, and Connors et al. (2012) evidenced a retention rate of 15.8%.

Regarding follow-up length, it was more common to conduct a single follow-up session (*n* = 7; Crane & Eckhardt, 2013; Lila et al., 2018, 2020; Murphy et al., 2017; Musser et al., 2008; Romero-Martínez et al., 2019; Santirso et al., 2020b), followed by three follow-up sessions, six and 12 months after (*n* = 3; Murphy et al., 2017, 2018; Stuart et al., 2013) and two follow-up sessions—six and 12 months after (*n* = 1; Alexander et al., 2010). Regarding single follow-up sessions, six studies (Crane & Eckhardt, 2013; Lila et al., 2018, 2020; Murphy et al., 2017; Musser et al., 2008; Santirso et al., 2020b) carried out a session 6 months after the PIPs’ completion and one study (Romero-Martínez et al., 2019) conducted it 9 months after.

### Characteristics of the Intervention Programs

Fourteen programs were delivered in the community and one in prison (Connors et al., 2012). However, most participants were referred to the intervention by the court (*n* = 12; Alexander et al., 2010; Connors et al., 2012; Crane & Eckhardt, 2013; Kistenmacher & Weis, 2008; Lila et al., 2018, 2020; Murphy et al., 2012; Romero-Martínez et al., 2019; Santirso et al., 2020b; Scott et al., 2011; Stuart et al., 2013; Zalmanowitz et al., 2013). Three different types of MIT were identified: the MI (Alexander et al., 2010; Crane & Eckardt, 2013; Crane &Eckhardt, 2013; Kistenmacher & Weiss, 2008; Murphy et al., 2012, 2018; Musser et al., 2008; Stuart et al., 2013), the Individualized Motivational Plan (IMP; Lila et al., 2020, 2018; Romero-Martínez et al., 2019; Santirso et al., 2020b; Zalmanowitz et al., 2013), and techniques for improving motivation (Connors et al., 2012; Scott et al., 2011).

MIT was used to: improve PIPs’ attendance (Scott et al., 2011), empathy and emotional decoding (Lila et al., 2020; Romero-Martínez et al., 2019), motivation for change (Connors et al., 2012; Murphy et al., 2012), increase treatment compliance and decrease recidivism rates (Crane & Eckhardt, 2013), change beliefs about violent behavior (Kistenmacher & Weiss, 2008), improve working therapeutic alliance (Lila et al., 2020; Musser et al., 2008; Santirso et al., 2020b), promote global functioning (Zalmanowitz et al., 2013), alcohol education (Stuart et al., 2013), and improve the overall effectiveness of PIPs (Lila et al., 2020). 10 manuscripts used MIT as a complement of a standard PIP, and five studies used MIT isolated.

Among the MIT programs as a complement, five intervention programs were based on MI (Alexander et al., 2010; Crane & Eckardt, 2013; Crane & Eckhardt, 2013; Murphy et al., 2012; Stuart et al., 2013), five on IMP (Lila et al., 2018, 2020; Romero-Martínez et al., 2019; Santirso et al., 2020b; Zalmanowitz et al., 2013), and one used techniques for improving motivation (Scott et al., 2011). The number of MIT sessions ranged from one (*n* = 2; Crane & Eckhardt, 2013; Stuart et al., 2013) to eight (*n* = 1; Lila et al., 2018). The most common number of sessions was two (Murphy et al., 2012; Musser et al., 2008; Zalmanowitz et al., 2013). The duration of each session ranged from 45 (*n* = 3; Crane & Eckhardt, 2013; Murphy et al., 2012; Musser et al., 2008) to 90 minutes (*n* = 1; Stuart et al., 2013), and most of them were conducted individually (*n* = 8; Crane & Eckhardt, 2013; Lila et al., 2020; Murphy et al., 2012; Musser et al., 2008; Romero-Martínez et al., 2019; Santirso et al., 2020b; Stuart et al., 2013; Zalmanowitz et al., 2013).

When MIT was used as an isolated treatment, four intervention programs used MI techniques (Kistenmacher & Weiss, 2008) and one used techniques for improving motivation (Connors et al., 2012), and the number of sessions ranged between two (*n* = 1; Kistenmacher & Weiss, 2008) and 32 (Connors et al., 2012). The duration of each session varied between 50 (Kistenmacher & Weiss, 2008) and 180 minutes (Connors et al., 2012). Two interventions were delivered individually (Kistenmacher & Weiss, 2008; Murphy et al., 2018), one in group (Alexander et al., 2010), and one in both modalities (Connors et al., 2012). Murphy et al. compared an individual MIT condition with a group MIT condition. Most PIPs used Cognitive-Behavioral Therapy (CBT; *n* = 6; Lila et al., 2018, 2020; Murphy et al., 2012; Musser et al., 2008; Romero-Martínez et al., 2019; Santirso et al., 2020b). Scott et al. (2011) and Zalmanowitz et al. (2013) adopted a pro-feminist approach. Although Crane and Eckhardt (2013) and Stuart et al. (2013) also included a PIP to complement MI intervention, the PIP’s approach/model was not specified. PIPs ranged between ten (Scott et al., 2011) and 35 weeks (Lila et al., 2018, 2020; Romero-Martínez et al., 2019; Santirso et al., 2020b), excluding MI sessions.

### Main Findings of the Analyzed Studies

#### Measurement Characteristics

All studies evaluated MITs and PIPs' efficacy using self-report instruments (*n* = 15) in two different ways: by comparing the results of the different measures in pre- and post-test (*n* = 1; Connors et al., 2012) or by comparing the experimental group—in which MI had been performed—with the control group (*n* = 14; Alexander et al., 2010; Crane & Eckhardt, 2013; Kistenmacher & Weiss, 2008; Lila et al., 2018, 2020; Murphy et al., 2012, 2017, 2018; Musser et al., 2008; Santirso et al., 2020b; Scott et al., 2011; Stuart et al., 2013; Romero-Martínez et al., 2019; Zalmanowitz et al., 2013). Four studies (Crane & Eckhardt, 2013; Lila et al., 2018, 2020; Murphy et al., 2017) also used official records to measure recidivism rates.

#### Intervention’s Outcomes

Intervention outcomes are presented according to the MIT modality, that is, as a complement to PIPs or as an isolated intervention.

#### MIT as a Complement to PIPs

Studies suggest that significant treatment gains were observed in MIT intervention in different outcomes. Participants who have been submitted to MIT evidenced dropout rates varying between 15% (Kistenmacher & Weiss, 2008) and 23% (Lila et al., 2018), and completion rates ranged between 84.2% (Scott et al., 2011) and 90% (Murphy et al., 2017). Those who attended only a standard intervention (SI) showed dropout rates ranging from 26.3% (Lila et al., 2020) and 53.5% (Scott et al., 2011) and completion rates between 46.5% (Crooks et al., 2011) and 85.6% (Murphy et al., 2018). In general, results revealed higher completion rates among individuals who attended MIT than those who attended SI (e.g., Crane & Eckhardt, 2013; Santirso et al., 2020b). Besides, on average, participants in the MIT condition attended more sessions than those in the SI condition (e.g., Lila et al., 2018). However, Musser et al. (2008) found no differences in treatment attendance between both conditions.

Studies that examined the stage of change (Murphy et al., 2020; Lila et al., 2018, 2020) found that MIT participants were in an advanced stage of change at the end of the intervention compared to participants of SI. However, Musser et al. (2008) found no differences between MIT participants and SI participants on readiness to change.

None of the studies that assessed official recidivism (Crane & Eckhardt, 2013; Lila et al., 2018, 2020) revealed significant differences between participants from MIT and SI conditions. However, Stuart et al. (2013) found lower perpetration of severe physical and psychological violence among MIT participants, and Lila et al. (2018, 2020) found lower physical violence perpetration and higher reduction in IPV risk recidivism for MIT participants.

Studies also analyzed other outcomes. For example, *working and therapeutic alliance* was analyzed in three studies (Lila et al., 2020; Musser et al., 2008; Santirso et al., 2020b). Results revealed that participants who received PIP in combination with MI showed higher working alliance than those who received only SI, regardless of the intervention moment. *Responsibility for abuse* was assessed in two studies (Musser et al., 2008; Santirso et al., 2020b), and participants submitted to MIT showed higher responsibility for abuse than those who received SI. *Empathy and emotional decoding* were also assessed in two studies (Lila et al., 2020; Romero-Martínez et al., 2019), with perpetrators who received MIT combined with PIP becoming more accurate in decoding emotional facial signals and improving their perspective-taking after the intervention program. One study assessed reductions in *alcohol use* (Stuart et al., 2013), with MIT participants reporting fewer alcohol consumption and greater abstinence at follow-up than participants in SI. Zalmanowitz et al. (2013) found lower levels of *distress* in individuals who received MIT plus PIP. At last, in a study by Musser et al. (2008), MIT participants showed an enhancement in *treatment engagement* and *help-seeking* behaviors, displayed more *constructive behavior* during PIP sessions, articulated greater *perceived value of treatment*, and completed more *CBT homework* than SI participants.

Despite the results mentioned above, outcomes might vary according to participants’ stage of change and readiness to change at intake. For example, Murphy et al. (2012) noticed that early-stage clients (those endorsing precontemplation, contemplation, or preparation before intake) progressed forward (1.31 stages in MIT condition vs. .17 stages in SI), while participants endorsing maintenance at pre-test regressed (1.44 stages in MIT condition vs. .45 stages in SI). Concerning *physical violence and partner assault rates* after treatment, participants with lower levels of pre-treatment contemplation submitted to MIT reported less physical violence and a reduction in partner assault (Lila et al., 2018; Murphy et al., 2012). Studies that examined the effect of stage of change on working alliance (Lila et al., 2020; Murphy et al., 2012; Santirso et al., 2020b) found that participants in the MIT condition who reported higher contemplation of change developed stronger working alliance than those in the SI condition. Concerning readiness to change, according to Crane and Eckhardt’s (2013) study, MIT participants low in readiness to change attended, on average, more sessions than control participants low in readiness. Regarding *completion rates*, resistant participants assigned to MIT were 10.13 times more likely to complete treatment than resistant participants assigned to SI and 4.93 times more likely to complete treatment than non-resistant participants (Scott et al., 2011). Concerning *dropout rates,* Scott et al. (2011) found that resistant clients in SI evidenced the highest dropout rate (53.5%), followed by non-resistant clients (38.9%) and resistant clients in MIT (15.8%).

#### Isolated MIT’s

Dropout rates from MIT intervention vary between 10% (individual MIT; Murphy et al., 2017) and 38% (group MIT; Murphy et al., 2017). MIT participants also showed higher rates of *treatment attendance* than the control group (Kistenmacher & Weiss, 2008). Also, MIT participants evidenced higher attendance rates when the intervention was carried out individually than in group (100% attendance in individual MIT condition; 71% in group MIT condition; Murphy et al., 2017). Results regarding *the stage of change* revealed that the MIT participants demonstrated higher pre-to-post increases in action and contemplation stages than controls (Kistenmacher & Weiss, 2008). Connors et al. (2012) also found an improvement in motivation for change among MIT participants. However, Alexander et al. (2010) found no differences in readiness to change between MIT condition and controls.

Concerning *physical violence*, Alexander et al. (2010) found a decrease in physical aggression but not in psychological aggression reported by partners among MIT participants. Murphy et al. (2018) also found significant reductions in violence perpetration in individuals from MIT condition. Murphy et al. (2017) found contradictory findings with a reduction of violence in the MIT group condition and an increase of individual violence in the MIT condition reported by partners. MIT participants also revealed improvements in thinking about changing their behavior and lower outsourcing of guilt (Kistenmacher & Weiss, 2008), greater acceptance of responsibility (Connors et al., 2012; Kistenmacher & Weiss, 2008), positive changes in attitudes, reduction in feelings of jealousy, anger, and dependency, improvement in their ability to dispute their cognitive distortions regarding their violence, and improvement in their ability to engage in perspective-taking and dealing with conflict (Connors et al., 2012). A study conducted by Murphy et al. (2018) also revealed that participants who completed the MIT intervention displayed greater awareness of alcohol problems, higher alcohol abstinence, and lower heavy drinking and illicit drug use.

In addition, Alexander et al. (2010) found that individuals less likely to change at intake were more likely to benefit from MIT intervention, while men more ready to change were more likely to benefit from CBT. Connors et al. (2012) also concluded that dropouts were more likely to report lower intake motivation to change than completers.

## Discussion

This systematic review aimed to analyze the incorporation of MIT (i.e., treatment alone and in combination with PIPs) in interventions with IPV perpetrators and its potential efficacy on perpetrators’ behavior and attitudes, motivation/readiness for change, and adherence to treatment/dropout rates. In addition, we aimed to understand the potential effect of the stage of change and readiness to change on MIT outcomes. Data from 15 manuscripts were included in this study. Results revealed that three types of MIT were used—MI, IMP, and techniques for improving motivation—, with three distinct aims: the use of MIT to assess the effectiveness of different intervention programs; the use of MIT in comparison with other interventions; and the use of MIT to assess their impact on certain variables/outcomes. In addition, MIT was used as a conjoint intervention with a standard PIP or as an isolated intervention.

The majority of the studies were conducted in North America, predominantly in the United States of America. This result is not surprising given that PIPs were primarily developed and implemented in the USA, where they quickly proliferated and became a popular penalty measure (e.g., Bowen, 2011). However, interestingly, some of the latest studies were conducted in Spain, highlighting the recent increase and proliferation of PIPs in this country (Ferrer-Perez et al., 2016). Nonetheless, this strategy may have influenced the country of origin because this systematic review search has been limited to English, Portuguese, and Spanish manuscripts.

Most intervention programs studied were delivered in community settings, despite participants being, in general, justice-involved ones (i.e., court-mandated). Only one program was delivered in a prison setting. This finding is particularly critical as research has suggested that MIT within prisons is a developing area showing effectiveness growth (Britt, 2014; McMurran, 2009). Thus, our results lead us to question whether MIT have been effectively applied in the intervention with incarcerated IPV perpetrators or whether the punitive culture that still characterizes correctional settings impacts the practice of MIT in this specific context.

In general, results from our systematic review follow those obtained by Santirso et al. (2020a), as IPV interventions that incorporated MIT were significantly more effective in increasing intervention dose and reducing dropout rates than interventions without the incorporation of MIT. We found that, with the implementation of MIT, participants demonstrated positive and greater effects in different outcomes than those who were only submitted to a standard PIP. MIT participants tend to reveal the greater perceived value of the treatment, greater commitment to intervention, greater recognition of violence and responsibility for their abusive behavior, and lower dropout rates. Significant improvements in motivation for change, empathy levels, and therapeutic alliance have also been verified after the implementation of MIT. Although the absence of variability regarding the setting in which intervention programs were implemented and the referral source prevent us from obtaining reliable conclusions, both community and prison programs seem to reveal positive outcomes regarding motivation improvement. These results are significant since, although individuals in both settings might enter the intervention program externally motivated, the improvement of internal motivation offered by the adoption of MIT during treatment programs seems crucial to an effective change.

Despite the use of different MIT types (i.e., MI, IMP, or techniques for improving motivation) and modalities (i.e., as a combined or as an isolated intervention), MIT proved to be effective with IPV perpetrators, mainly as a way to improve perpetrators' readiness and motivation for change and increase treatment adherence.

Regarding MIT as a complement to PIP (e.g., Crane & Eckhardt, 2013; Lila et al., 2020; Murphy et al., 2012; Romero-Martínez et al., 2019; Santirso et al., 2020b; Scott et al., 2011; Zalmanowitz et al., 2013), although the variability in MIT and PIP targets and length, MIT type adopted (i.e., MI, IMP, techniques for improving motivation), along with the MIT contents, and modality of MIT condition (i.e., individual or group), the number of sessions delivered, the type of MIT, and the intervention modality did not seem to influence the positive impact of the MIT on PIP. Indeed, a significant number of participants submitted to MIT completed the intervention (e.g., Crane & Eckhardt, 2013; Scott et al., 2011) and demonstrated high levels of commitment to the intervention (e.g., Lila et al., 2018; Murphy et al., 2012; Musser et al., 2008), completion rates (Santirso et al., 2020b) and working/therapeutic alliance (Lila et al., 2020; Santirso et al., 2020b). Studies also noticed an increase in responsibility for violence (Musser et al., 2008; Santirso et al., 2020b) and a decrease in violence perpetration (Lila et al., 2018, 2020; Stuart et al., 2013) and in IPV recidivism risk level (Lila et al., 2018, 2020) after MIT has been implemented. However, results regarding recidivism rates are ambiguous, and they cannot confirm the efficacy of MIT in reducing recidivism as none of the studies that used official recidivism revealed differences between the MIT and SI conditions.

Concerning MIT as an isolated treatment, although the variation among the studies in treatment targets, program length, modality, and MIT type, results also point to positive changes after the intervention, with MIT participants revealing greater responsibility assumption (Connors et al., 2012; Kistenmacher & Weiss, 2008), a decrease in violence perpetration (Alexander et al., 2010; Murphy et al., 2018), and changes in attitudes towards violence (Connors et al., 2012). Although both group and individual modalities revealed positive changes, Murphy et al. (2017) found higher attendance rates among participants in the individual condition and higher decreases in violence perpetration among group participants.

Despite the previously mentioned, it is important to stress that in both modalities, wide variability in MIT length and wide variation in the contents of MIT between studies, even when the same type of MIT was used, was observed. This variability prevents us from making reliable conclusions regarding the superiority of one modality over the other. However, both modalities revealed similar results regarding dropout rates, treatment attendance, and motivation improvement, all important factors for effective change. Overall, using MIT seems to be an important tool to prepare individuals for further interventions (when used as a complement) and improve intrinsic motivation for change, breaking the resistance to change and improving intervention adherence. Therefore, both modalities seem to be valid, and the choice for one or the other should be considered in facing the specificities of each case.

Although the results of our systematic review favor MIT strategies, most studies have short follow-up periods (between 6 and 12 months) and rely solely on perpetrators’ self-reports, and the use of self-report measures among IPV perpetrators still presents some concerns (Babcock et al., 2004). In addition, although some studies reported that MIT participants revealed a positive evolution in the stage of change through the intervention, the stage of change and readiness to change at the beginning of the intervention might have a differential impact on the outcomes. As referred by previous research (e.g., Farbring & Johnson, 2008; Miller & Rollnick, 2012), MIT demonstrates greater efficacy (e.g., reduction in violence perpetration and partner assault) with more ambivalent and change-resistant participants (e.g., Alexander et al., 2010; Crane & Eckhardt, 2013; Lila et al., 2018; Murphy et al., 2012); that is, with individuals in the early stages of change at the intake. Besides, individuals higher in contemplation (i.e., ambivalent towards intervention) tend to reveal greater working and therapeutic alliance (Lila et al., 2020; Murphy et al., 2012; Santirso et al., 2020b).

Analyzing dropout and completion rates, the efficacy of MIT is visible as most studies revealed low dropout rates for MIT participants (e.g., Crane & Eckhardt, 2013; Lila et al., 2018; Lila et al., 2020; Murphy et al., 2018). These results are significant as the non-completion of intervention programs is considered a significant obstacle to treatment success (e.g., McMurran et al., 2010), individuals’ welfare, and public safety (Olver et al., 2011). Indeed, dropout is a predictor of IPV recidivism (e.g., Lauch et al., 2017; Lila et al., 2019; Olver et al., 2011), and many variables that predict treatment dropout also predict IPV recidivism (e.g., Cattaneo & Goodman, 2005; Stith et al., 2004). As such, efforts to improve IPV perpetrators’ motivation should be seen as a requirement for commitment to the intervention and motivation for change. Since MIT is able to motivate IPV perpetrators to attend, stay committed to treatment, and complete the intervention, it will consequently reduce dropout and, therefore, recidivism rates. This is verifiable in the study of Scott et al. (2011), which demonstrated that resistant clients who were submitted to MIT evidenced lower rates of dropout (15.8%) in comparison with the ones who just participated in a standard PIP (53.5%).

### Strengths and Limitations of This Review

This systematic review allows us to understand the potential benefits of including MIT in IPV perpetrators' programs to increase motivation to change and treatment adherence and decrease dropout and recidivism rates. Besides, interventions that integrated MIT seem more effective than interventions without MIT.

Despite the contributions, some limitations should be mentioned. The main limitation identified was the high prevalence of studies conducted in the USA (compared to other countries) and the absence of studies in languages other than English and Spanish, which would allow a greater understanding of the approach and method utilized in other countries to investigate MIT and PIPs. Also, the information provided by the studies on dropout and completion rates may have conditioned a deeper knowledge of this matter since some studies did not specify all that information or only mentioned one type of rate (either dropout rates or completion rates). In addition, although the inclusion of a wide variety of studies allows differentiating this systematic review from other systematic reviews and meta-analyses, it made it difficult to gather a deeper comprehension of the best/more effective way to integrate MIT. More specifically, the variability in the designs used, the treatment targets, number of sessions held (ranging between one and eight sessions), duration of each session (varies between 50 and 180 minutes), type of MIT (MI, IMP, techniques for improving motivation), contents of MIT, and format (individual vs. group) prevent us from making reliable conclusions regarding MIT efficacy with IPV perpetrators. Further studies should consider these issues to paint an overall picture of MIT efficacy.

## Conclusion: Future Research

The main objective of this systematic review was to understand the influence of MIT on the efficacy of intervention programs for IPV perpetrators. This study enables us to establish the positive impact MIT’s integration has on perpetrators’ motivation to change, commitment, dropout, and efficacy of PIPs. Besides, this systematic review corroborated the great pertinence of this technique in this specific context. Still, developing systematic reviews, meta-analyses, and other studies analyzing MIT remains essential. Specifically, more research is needed to better understand the impact of MIT on the efficacy of PIPs through randomized controlled trials with longer follow-up periods to adequately assess the persistence of change (e.g., Santirso et al., 2020a). Besides, to reduce dada bias, self-report and official data should be included to properly assess the efficacy of MIT in the recurrence of IPV. As the follow-up length in most studies is 6 months, with a large follow-up period of 12 months, it is recommended that further studies include more extended follow-up periods to better assess the persistence of change. Carrying out longitudinal approaches should also be considered, as they will generate rich and in-depth knowledge (Tables 3 and 4).

**Table 3.** Key Findings of the Systematic Review.

• The present systematic review concluded that there is a robust research trend in the analysis of the effectiveness of MI
• Use of MI for multiple purposes: to assess the effectiveness of certain programs and/or instruments; to compare MI with other interventions; and to assess the improvement of other variables (e.g., alcohol consumption, therapeutic alliance)
• 15 studies were part of this systematic review, all of them pointing to the effectiveness of MI with IPV perpetrators
• MIT have shown a significant influence on program adherence, dropout, and recidivism

**Table 4.** Implications for Research, Practice, and Policy.

Implications for research	Implications for practices and policy
• More randomized clinical trials are necessary to evaluate specific strategies to increase the effectiveness of PIPs	• IPV perpetrators’ motivation to change should be appropriately assessed in order to enhance the effectiveness of the intervention
• more research is needed to better understand the impact of MIT on PIPs effectiveness	• there is good evidence of MI strategies in increasing the effectiveness of PIPs
• further research should include both self-report and official data to assess MIT effectiveness on IPV recidivism	• Efforts to improve IPV perpetrators’ motivation should be included in PIPs by incorporating MIT as it might increase adherence and motivation to change and therefore reduce recidivism rates
	• Governments should provide and support continued investment in PIPs that address IPV perpetrators’ motivation to change
	• more public services and agencies for IPV perpetrators that want to change should be provided

